# Pharmacological profile of the neuropeptide S receptor: Dynamic mass redistribution studies

**DOI:** 10.1002/prp2.445

**Published:** 2018-12-03

**Authors:** Chiara Ruzza, Federica Ferrari, Remo Guerrini, Erika Marzola, Delia Preti, Rainer K. Reinscheid, Girolamo Calo

**Affiliations:** ^1^ Department of Medical Sciences Section of Pharmacology National Institute of Neuroscience University of Ferrara Ferrara Italy; ^2^ Department of Chemical and Pharmaceutical Sciences and LTTA University of Ferrara Ferrara Italy; ^3^ Institute of Pharmacology and Toxicology Jena University Hospital Friedrich Schiller University Jena Jena Germany; ^4^ Institute of Physiology I University Hospital Münster University of Münster Münster Germany

**Keywords:** dynamic mass redistribution assay, in vitro pharmacology, label‐free assay, neuropeptide S, neuropeptide S receptor

## Abstract

Neuropeptide S (NPS) is the endogenous ligand of the neuropeptide S receptor (NPSR). NPS modulates several biological functions including anxiety, wakefulness, pain, and drug abuse. The aim of this study was the investigation of the pharmacological profile of NPSR using the dynamic mass redistribution (DMR) assay. DMR is a label‐free assay that offers a holistic view of cellular responses after receptor activation. HEK293 cells stably transfected with the murine NPSR (HEK293_mNPSR_) have been used. To investigate the nature of the NPS‐evoked DMR signaling, FR900359 (Gq inhibitor), pertussis toxin (Gi inhibitor), and rolipram (phosphodiesterase inhibitor) were used. To determine the pharmacology of NPSR, several selective ligands (agonists, partial agonists, antagonists) have been tested. NPS, through selective NPSR activation, evoked a robust DMR signal with potency in the nanomolar range. This signal was predominantly, but not completely, blocked by FR900359, suggesting the involvement of the Gq‐dependent signaling cascade. NPSR ligands (agonists and antagonists) displayed potency values in DMR experiments similar, but not identical, to those reported in the literature. Furthermore, partial agonists produced a higher efficacy in DMR than in calcium experiments. DMR can be successfully used to study the pharmacology and signaling properties of novel NPSR ligands. This innovative approach will likely increase the translational value of in vitro pharmacological studies.

**Abbreviations**
BSA: bovine serum albumin
cAMP: cyclic AMP
DMEM: Dulbecco's Modified Eagle's Medium
DMR: Dynamic Mass Redistribution
FR900359: [(1R)‐1‐[(3S,6S,9S,12S,18R,21S,22R)‐21‐acetamido‐18‐benzyl‐3‐[(1R)‐1‐methoxyethyl]‐4,9,10,12,16‐pentamethyl‐15‐methylidene‐2,5,8,11,14,17,20‐heptaoxo‐22‐propan‐2‐yl‐1,19‐dioxa‐4,7,10,13,16‐pentazacyclodocos‐6‐yl]‐2‐methylpropyl] (2S,3R)‐3‐hydroxy‐4‐methyl‐2‐(propanoylamino)pentanoate
GPCR: G protein‐coupled receptor
HBSS: Hank's Balanced Salt Solution
HEK293: Human Embryonic Kidney 293 cells
HEPES: 4‐(2‐hydroxyethyl)‐1‐piperazineethanesulfonic acid
NECA: (2S,3S,4R,5R)‐5‐(6‐aminopurin‐9‐yl)‐N‐ethyl‐3,4‐dihydroxyoxolane‐2‐carboxamide
NOP: Nociceptin/Orphanin FQ Receptor
NPS: Neuropeptide S
NPSR: Neuropeptide S Receptor
PTX: Pertussis Toxin
SHA68: N‐[(4‐fluorophenyl)methyl]‐3‐oxo‐1,1‐di(phenyl)‐5,6,8,8a‐tetrahydro‐[1,3]oxazolo[4,3‐c]pyrazine‐7‐carboxamide

## 1 INTRODUCTION

Neuropeptide S (NPS, primary sequence in humans: SFRNGVGTGMKKTSFQRAKS) was identified in 2002 as the endogenous ligand of the previously orphaned G protein‐coupled receptor (GPCR) GPR154, now referred to as neuropeptide S receptor (NPSR), using the reverse pharmacological approach. In 2004, an elegant study by Xu et al described, for the first time, some functional features of the NPS/NPSR system.^1^ NPSR is a GPCR showing moderate homology to other members of the GPCR family.^2^ The in vitro pharmacology of the human and mouse NPSR has been mainly studied in heterologous expression systems. These studies showed that NPS increases both intracellular calcium levels and cAMP accumulation with EC_50_ values in the low nanomolar range. This indicates that NPSR can signal via both Gq and Gs pathways to increase cellular excitability.^1^, ^3^ In vivo, NPS has been shown to modulate several biological functions in rodents including stress, anxiety, social behavior, locomotor activity, wakefulness, food intake and gastrointestinal functions, memory processes, pain, and drug abuse (for reviews see 4 and 5).

Up to now, NPSR ligands have been characterized in vitro using single end‐point assays, that is, calcium mobilization and cAMP accumulation. This approach might be reductionist providing incomplete pharmacological profiles and eventually biasing the translatability from medicinal chemistry to the biological level. Label‐free assays now offer the possibility to have, in a noninvasive manner, a holistic view of cellular responses after receptor activation. Label‐free assays use special biosensors (electron‐conducting or light‐diffracting plates) to translate the receptor‐dependent holistic cellular response to physical parameters such as variations of impedance or modulations of wavelength shift of an incident light in real time. The dynamic mass redistribution (DMR) assay is a label‐free approach based on an optical biosensor technology.^6^, ^7^ Using a resonant waveguide grating, DMR measures changes in the refractive index of the bottom portion of the cell layer. Several intracellular events can lead to changes in the cells refractive index, that is, protein recruitment, receptor internalization and recycling, second messenger alternation, cytoskeletal remodeling, and cell adhesion changes.^8^ DMR has been already applied to study the pharmacological properties of new ligands acting at various GPCRs, such as histamine H_1_,^9^, ^10^ β_2_ adrenergic,^11^, ^12^ muscarinic M_3_,^13^ purinergic P2Y,^14^ formyl peptide,^15^ and protease‐activated^16^, ^17^ receptors. Classical opioid^18^, ^19^ and the nociceptin/orphanin FQ peptide (NOP)^20^ receptors were also investigated in DMR studies. Additionally, the DMR assay, together with different biochemical tools, has been successfully used for GPCRs signaling deconvolution studies.^7^, ^9^, ^21^, ^22^

The present study investigates the pharmacological profile and signaling of the murine NPSR expressed in human embryonic kidney 293 (HEK293) cells using the DMR assay. Importantly, this isoform of the receptor and these cells have been chosen based on the fact that all the compounds investigated in the present study have been previously characterized in calcium mobilization studies performed on HEK293_mNPSR_ cells, thus making possible a direct comparison of the DMR and calcium mobilization results.

## 2 MATERIALS AND METHODS

### 2.1 Drugs and reagents

NPS fragments (NPS(2‐20), NPS(3‐20), NPS(1‐6), NPS(1‐10)^23^) and a large series of NPS‐related peptides ([Ala^7^]NPS, [Ala^3^]NPS,^23^ [Bip^2^]NPS,^24^ [D‐Ala^5^]NPS and [Aib^5^]NPS,^25^ [D‐Cys(^*t*^Bu)^5^]NPS,^26^ [D‐Val^5^]NPS,^27^ [^*t*^Bu‐D‐Gly^5^]NPS,^28^, ^29^ [D‐Pen‐S‐p(^*t*^BuBzl)^5^]NPS^30^) were assayed. In addition, the tetrabranched derivative of NPS PWT1‐NPS^31^ and the nonpeptide NPSR antagonist SHA 68^32^, ^33^ were included in the study. NPS and its derivatives were synthesized in house according to published methods. SHA 68 was purchased from Tocris Bioscience (Bristol, UK). Pertussis toxin (PTX) was from List Biological Laboratories, Inc. (Campbell, CA, USA). 5′‐N‐ethylcarboxamidoadenosine (NECA), rolipram, brilliant black, bovine serum albumin (BSA), and 4‐(2‐ hydroxyethyl)‐1‐piperazineethanesulfonic acid (HEPES) were from Sigma Aldrich (St. Louis, MO, USA). FR900359 (UBO‐QIC) was from Prof. Kostenis laboratory (University of Bonn, Germany). All cells culture media and supplements were from Lonza (Basel, Switzerland). NPS and its derivatives as well as PTX were dissolved in ultrapure water (1 mmol L^−1^). SHA 68, FR900359, rolipram, and NECA were dissolved in dimethyl sulfoxide (DMSO, 10 mmol L^−1^ stocks). Stock solutions were kept at −20°C until use. Serial dilutions were made in assay buffer (Hanks’ Balanced Salt solution (HBSS)/HEPES 20 mmol L^−1^, containing 0.01 % BSA and 0.1% DMSO).

### 2.2 Cells

HEK293 cells stably expressing the murine NPSR receptor (HEK293_mNPSR_) were described previously.^3^ Wild‐type HEK293 cells were used as a control. Cells were maintained in Dulbecco's Medium (DMEM) supplemented with 10% fetal bovine serum, 100 U/mL penicillin, 100 μg/mL streptomycin and, 2 mmol L^−1^ l‐glutamine. The medium was complemented with 100 mg/L hygromycin for HEK293_mNPSR_. Cells were cultured at 37°C in 5% CO_2_ humidified air.

### 2.3 Dynamic mass redistribution assay

Confluent cells were subcultured using trypsin/EDTA and used for experiments. Cells were seeded at a density of 20 000 cells/well in 30 μL into fibronectin‐coated Enspire™‐LC 384‐wells plates and cultured for 20 hours to form a confluent monolayer. On the day of the experiment, cells were manually washed twice and maintained with assay buffer for 90 minutes before DMR experiments. DMR was monitored in real time with a temporal resolution of 22 seconds throughout the assay. Experiments were performed at 37°C, using an EnSight Multimode Plate Reader (PerkinElmer), that uses the Corning^®^ Epic^®^ Technology to measure the DMR signal. Agonism protocol: a 5‐minute baseline was first established, followed by adding compounds manually in a volume of 10 μL and recording compounds‐triggered DMR signal for 60 minute. Antagonism protocol: antagonists were added manually 25 minute before reading the 5‐minute baseline. After baseline establishment, NPS was injected and DMR signal was recorded for 60 minute. Antagonist properties of ligands were measured by assessing the concentration‐response curve to NPS in the absence and in presence of a fixed concentration of compound. FR900359 was added 60 minute before NPS, rolipram was incubated for 90 minute before NPS, while PTX was added 24 hours before NPS. Maximum picometer (pm) modifications (peak measured at 60 minute time point) were used to determine agonist response after baseline normalization.

### 2.4 Calcium mobilization assay

Cells were seeded at a density of 50 000 cells/well in 100 μL into poly‐d‐lysine coated 96‐well black, clear‐bottom plates. The following day, cells were incubated with medium supplemented with 2.5 mmol L^−1^ probenecid, 3 μmol L^−1^ of the calcium sensitive fluorescent dye Fluo‐4 AM and 0.01% pluronic acid, for 30 minute at 37°C. Following this, the loading solution was aspirated and 100 μL of HBSS supplemented with 20 mmol L^−1^ HEPES, 2.5 mmol L^−1^ probenecid and 500 μmol L^−1^ Brilliant Black were added. Cell culture and drug plates were placed into a fluorimetric imaging plate reader (FlexStation II, Molecular Devices, Sunnyvale, CA) and fluorescent changes were measured. On‐line additions were carried out in a volume of 50 μL/well. To facilitate drug diffusion into the wells, the present studies were performed at 37°C and three cycles of mixing (25 μL from each well moved up and down three times) were performed immediately after FR900359 injection to the wells. FR900359 was injected into the wells 24 minute before adding NPS.

### 2.5 Data analysis and terminology

All data were analyzed using Graph Pad Prism 6.0 (La Jolla, CA, USA). Concentration‐response curves were fitted using the four parameters log logistic equation. Data are expressed as mean ± SEM of n experiments performed in duplicate and were analyzed using one‐ or two‐way analysis of variance (ANOVA) followed by Dunnett's or Tukey's test for multiple comparisons wherever appropriate. Agonist potency was expressed as pEC_50_, which is the negative logarithm to base 10 of the agonist molar concentration that produces 50% of the maximal possible effect of that agonist. Antagonists potencies were assayed at single concentrations against the concentration‐response curve to NPS and their pA_2_ was derived using the following equation: pA_2_ = log(CR − 1)  − log[A], where CR is the ratio between agonist potency (EC_50_) in the presence and in absence of antagonist and [A] is the molar concentration of antagonist.

## 3 RESULTS

### 3.1 DMR effects of NPS

**Figure 1:**
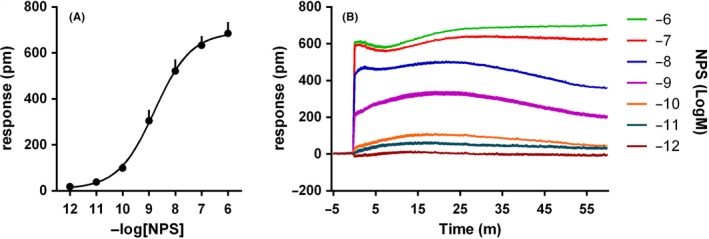
Concentration‐response curve to NPS. Sigmoidal curve is shown in (A), while representative raw DMR traces are displayed in (B). Data are the mean ± SEM of six experiments performed in duplicate

**Figure 2:**
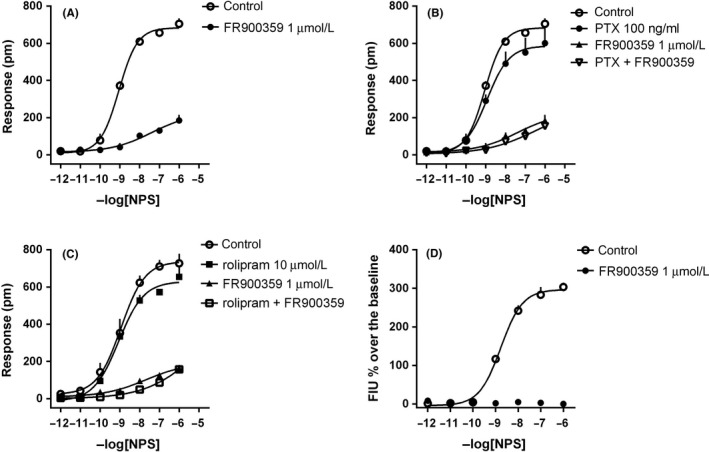
DMR assay, concentration‐response curves to NPS in the absence and presence of FR900359 (A), PTX and FR900359 + PTX (B), rolipram and FR900359 + rolipram (C). Calcium mobilization assay, concentration‐response curves to NPS in the absence and presence of FR900359 (D). Data are the mean ± SEM of at least three experiments performed in duplicate

In HEK293_mNPSR_ cells, NPS evoked a robust concentration‐dependent DMR response, with pEC_50_ of 8.78 (8.22‐9.34) and maximal effect of 690 ± 39 pm (Figure 1A). A representative trace of the NPS DMR signal is shown in Figure 1B. NPS was completely inactive in wild‐type HEK293 cells (Table 1). To analyze the biochemical nature of the NPS response, the peptide was tested in the absence and presence of the Gq inhibitor FR900359 (1 μmol L^−1^, Figure 2A), the Gi inhibitor PTX (100 ng mL^−1^, Figure 2B), and, with the aim to investigate the Gs dependent pathway, the phosphodiesterase inhibitor rolipram (10 μmol L^−1^, Figure 2C). FR900359 strongly, but not completely, reduced NPS effects in the DMR assay. Importantly, in parallel experiments performed in the same cells, FR900359 completely abolished the NPS stimulated calcium mobilization (Figure 2D). Two‐way ANOVA (treatment × assay) followed by the Tukey's multiple comparisons test, revealed that NPS 1 μmol L^−1^ + FR900359 evoked a significant response in the DMR but not in the calcium assay (treatment *F*_(1,8)_ = 28.87, assay *F*_(1,8)_ = 44.89, interaction *F*_(1,8)_ = 28.87; *P* < 0.05 vs vehicle). On the contrary, PTX did not modify NPS‐evoked DMR response, both alone and in combination with FR900359. Similar results were obtained with rolipram that did not change NPS effects neither when tested alone nor in presence of FR900359. Of note, at this concentration, rolipram was able to significantly enhance the DMR response of the adenosine receptor agonist NECA 10 μmol L^−1^ from 229 ± 18 to 464 ± 58 pm (*P* ˂ 0.05, according to Student's *t* test) in HEK293_mNPSR_ cells.

**Table 1:** Effects of high concentrations of ligands in HEK293 and HEK293_mNPSR_ cells

	HEK293 pm ± SEM	HEK293_mNPSR_ pm ± SEM
Buffer	33 ± 13	22 ± 3
NPS 1 μmol L^−1^	37 ± 14	670 ± 44^a^
NPS(2‐20) 10 μmol L^−1^	70 ± 10	782 ± 46^a^
NPS(1‐10) 10 μmol L^−1^	27 ± 6	683 ± 90^a^
NPS(3‐20) 10 μmol L^−1^	166 ± 14^a^	675 ± 54^a^
NPS(3‐20) 1 μmol L^−1^	41 ± 14	315 ± 17^a^
NPS(1‐6) 10 μmol L^−1^	25 ± 10	356 ± 48^a^
PWT1‐NPS 1 μmol L^−1^	245 ± 10^a^	796 ± 92^a^
PWT1‐NPS 0.1 μmol L^−1^	130 ± 37^a^	595 ± 48^a^
PWT1‐NPS 0.01 μmol L^−1^	42 ± 14	455 ± 45^a^
[Ala^7^]NPS 10 μmol L^−1^	115 ± 33	692 ± 15^a^
[Bip^2^]NPS 10 μmol L^−1^	191 ± 30^a^	761 ± 60^a^
[Bip^2^]NPS 1 μmol L^−1^	47 ± 16	577 ± 2^a^
[Ala^3^]NPS 10 μmol L^−1^	84 ± 15	574 ± 14^a^
[D‐Ala^5^]NPS 10 μmol L^−1^	55 ± 14	686 ± 50^a^
[Aib^5^]NPS 10 μmol L^−1^	54 ± 18	633 ± 8^a^
[D‐Cys(^*t*^Bu)^5^]NPS 1 μmol L^−1^	25 ± 8	387 ± 43^a^
NECA 10 μmol L^−1^	312 ± 28^a^	229 ± 18^a^
Carbachol 100 μmol L^−1^	480 ± 51^a^	393 ± 9^a^

a *P* < 0.05 vs buffer according to one‐way ANOVA followed by Dunnett's test for multiple comparisons (*F*_(18,38)_ = 30.42, HEK293; *F*_(18,38)_ = 25.12, HEK293_mNPSR_).

### 3.2 DMR effects of NPSR ligands

To further validate the DMR assay, responses to a large panel of NPSR agonists showing different values of potency and efficacy together with peptide and nonpeptide NPSR antagonists were investigated. All NPSR ligands that produced an effect in HEK293_mNPSR_ cells were tested in parallel experiments in wild‐type HEK293 cells. These results are summarized in Table 1. Specifically, NPS(3‐20) and [Bip^2^]NPS at 10 μmol L^−1^, but not at 1 μmol L^−1^, elicited a significant DMR response in wild‐type cells, thus for these compounds, 1 μmol L^−1^ was selected as highest concentration for further studies. PWT1‐NPS caused a significant DMR effect at 1 and 0.1 μmol L^−1^ in wild‐type cells, thus further experiments in HEK293_mNPSR_ were performed using 0.01 μmol L^−1^ as highest concentration for this ligand. All the remaining NPSR ligands tested at 10 μmol L^−1^ produced significant DMR responses in HEK293_mNPSR_ but not wild‐type HEK293 cells. As expected, NECA and carbachol, used as the positive controls, promoted similar effects in HEK293_mNPSR_ and wild‐type HEK293 cells.

**Figure 3:**
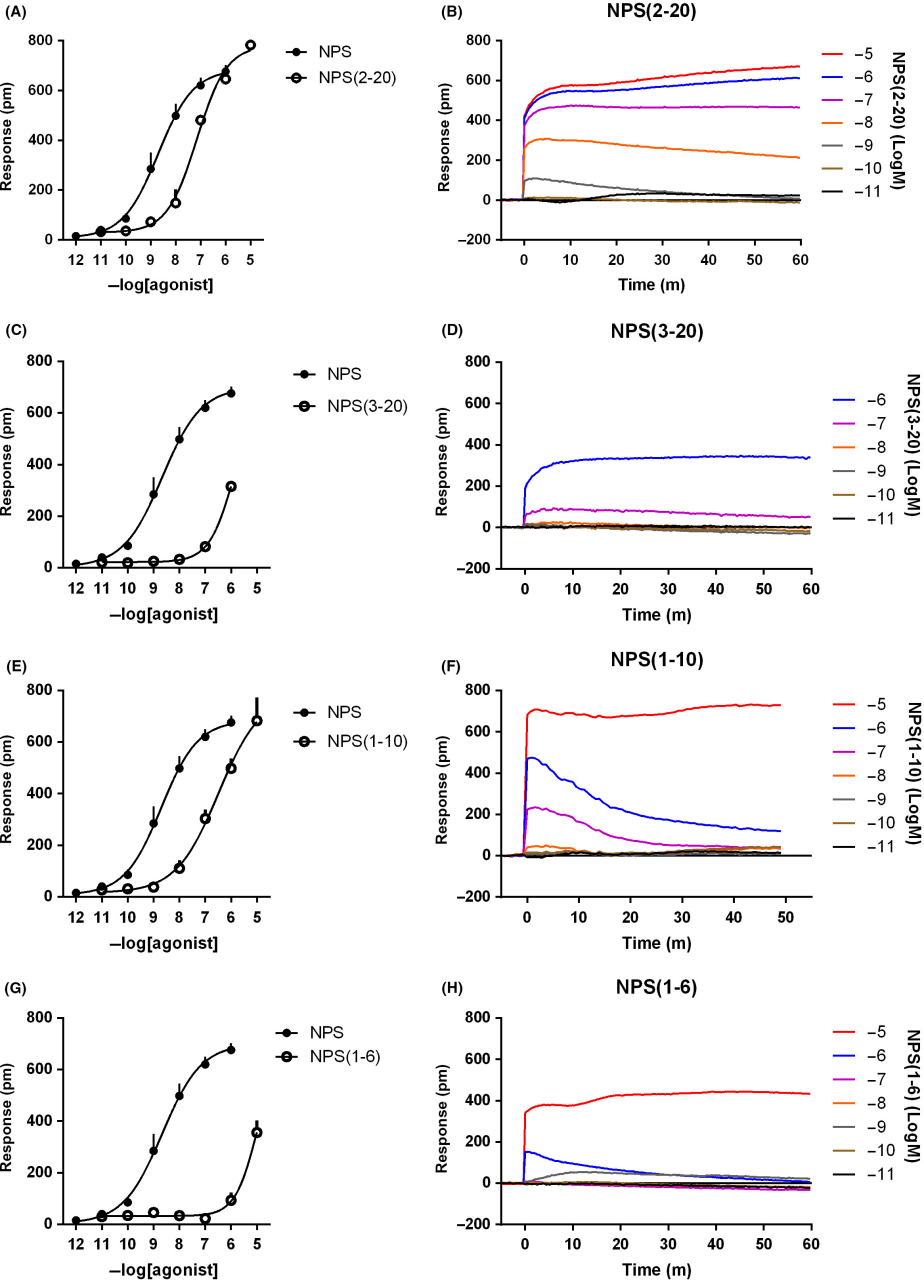
Concentration‐response curves to NPS (2‐20), NPS (3‐20), NPS (1‐10), and NPS (1‐6). Sigmoidal curves are shown in the left panels, while representative raw DMR tracings are displayed in the right panels. Data are the mean ± SEM of at least three experiments performed in duplicate

**Figure 4:**
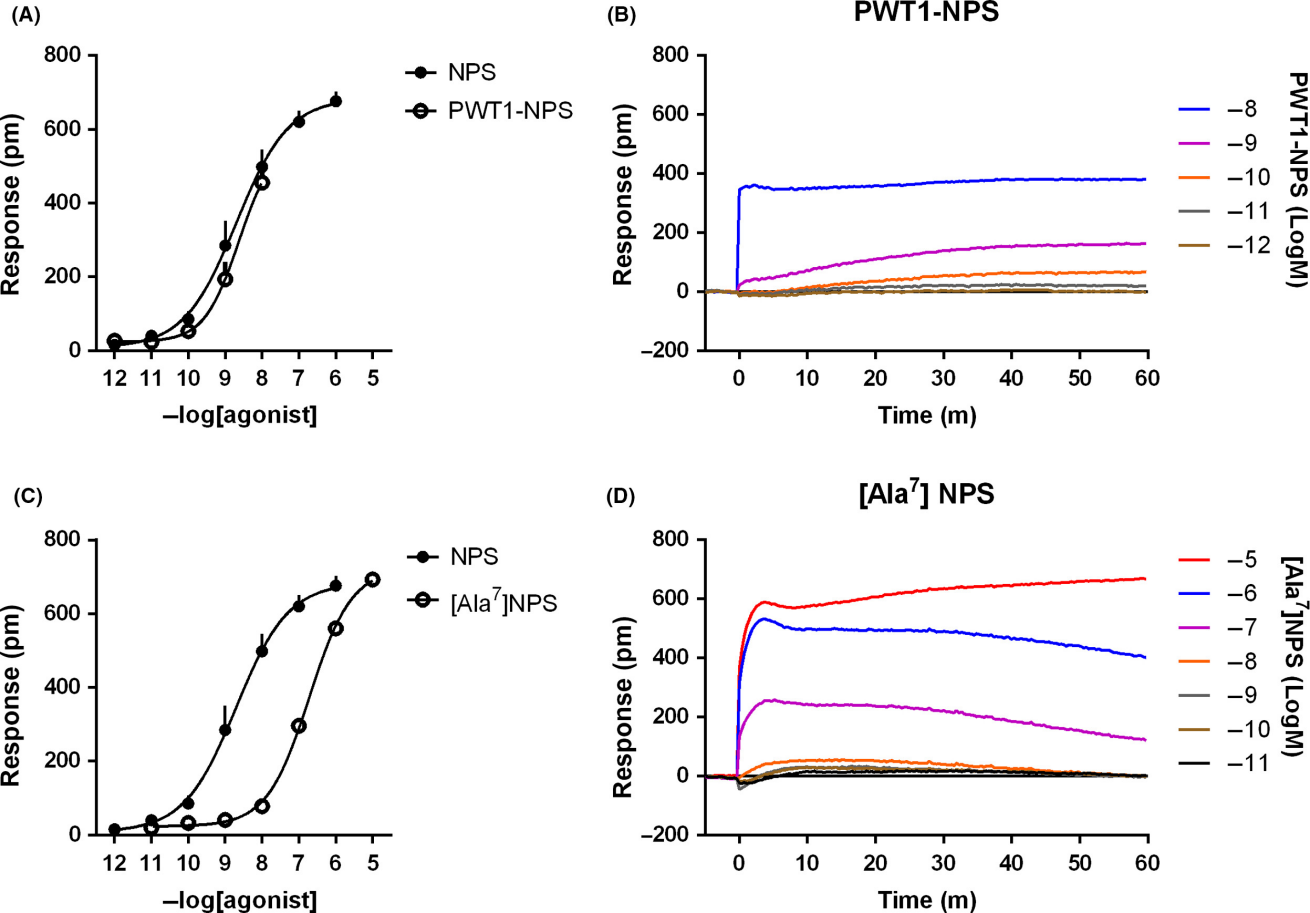
Concentration‐response curves to PWT1‐NPS and [Ala^7^]NPS. Sigmoidal curves are displayed in the left panels, while representative raw DMR tracings are shown in the right panels. Data are the mean ± SEM of at least three experiments performed in duplicate

In HEK293_mNPSR_ cells, NPS(2‐20) and NPS(1‐10) produced a concentration‐response curve with maximal effects similar to those elicited by NPS but with lower potency (pEC_50_ of 7.22 and 6.55, respectively). The fragments NPS(3‐20) and NPS(1‐6) elicited a DMR signal only at the higher concentration tested (Figure 3). PWT1‐NPS generated an incomplete concentration‐response curve, thus its potency and maximal effects could not be estimated. However, the effects of 1 and 10 nmol L^−1^ of PWT1‐NPS were virtually superimposable to those induced by the same concentrations of NPS. Finally, [Ala^7^]NPS was able to evoke a DMR response in HEK293_mNPSR_ with maximal effects similar to those of NPS but demonstrating ~20 fold loss in potency (Figure 4). Of note, the shape of the DMR responses promoted by the above‐mentioned NPSR ligands was similar to that recorded in response to NPS (right panels of Figures 3 and 4).

**Figure 5:**
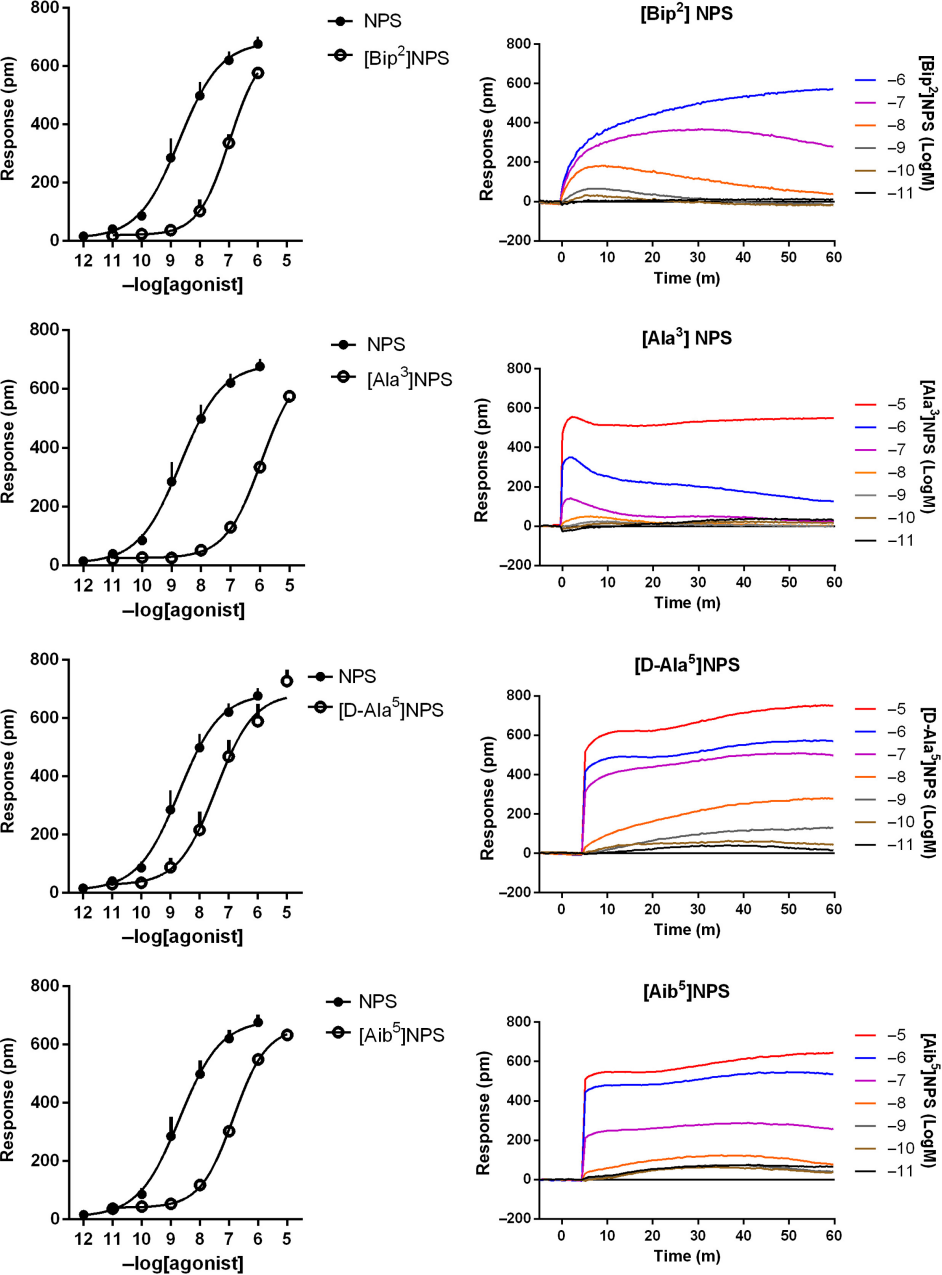
Concentration‐response curves to [Bip^2^]NPS, [Ala^3^]NPS, [D‐Ala^5^]NPS, and [Aib^5^]NPS. Sigmoidal curves are shown in the left panels, while representative raw DMR tracings are displayed in the right panels. Data are the mean ± SEM of at least three experiments performed in duplicate

Figure 5 shows the concentration‐response curves and representative DMR traces of NPS analogues reported in literature as NPSR partial agonists.^23^, ^24^, ^25^ All these compounds elicited DMR maximal effects similar to NPS. [Ala^3^]NPS displayed a very low potency that did not allow the determination of *E*_max_ and pEC_50_, while the other peptides were 30‐ 100‐fold less potent than NPS.

**Figure 6:**
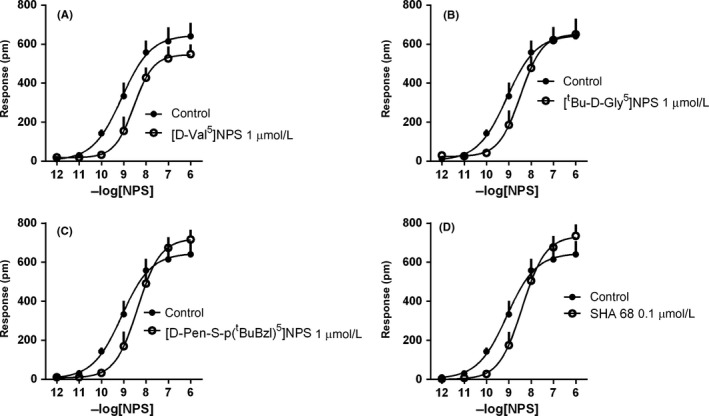
Concentration‐response curves to NPS in the absence and presence of [D‐Val^5^]NPS (1 μmol L^−1^, A), [^*t*^Bu‐D‐Gly^5^]NPS (1 μmol L^−1^, B), [D‐Pen‐S‐p(^*t*^BuBzl)^5^]NPS (1 μmol L^−1^, C), and SHA 68 (0.1 μmol L^−1^, D). Data are the mean ± SEM of three experiments performed in duplicate

The NPSR antagonists [D‐Cys(^*t*^Bu)^5^]NPS, [D‐Val^5^]NPS, [^*t*^Bu‐D‐Gly^5^]NPS, [D‐Pen‐S‐p(^*t*^BuBzl)^5^]NPS, and SHA 68 were tested alone and against the concentration‐response curve to NPS. These compounds did not produce any effect per se, with the exception of [D‐Cys(^*t*^Bu)^5^]NPS that at 1 μmol L^−1^ evoked a DMR response of 387 ± 43 pm (58% of NPS maximal effect). Of note, [D‐Cys(^*t*^Bu)^5^]NPS 1 μmol L^−1^ did not produce any effect in HEK 293 wild‐type cells (Table 1). [D‐Val^5^]NPS (1 μmol L^−1^), [^*t*^Bu‐D‐Gly^5^]NPS (1 μmol L^−1^), [D‐Pen‐S‐p(^*t*^BuBzl)^5^]NPS (1 μmol L^−1^), and SHA 68 (0.1 μmol L^−1^) promoted a rightward shift of the concentration‐response curve to NPS, without significant modifications of its maximal effects. The following pA_2_ values were derived from these experiments: 6.39 for [D‐Val^5^]NPS, 6.48 for [^*t*^Bu‐D‐Gly^5^]NPS, 6.60 for [D‐Pen‐S‐p(^*t*^BuBzl)^5^]NPS, and 7.59 for SHA 68 (Table 2 and Figure 6).

**Table 2:** Agonists potencies (pEC_50_), intrinsic activity (α), and antagonist potencies (pA_2_) of compounds tested in DMR assay in HEK293_mNPSR_ cells

	pEC_50_ (CL_95%_)	α ± SEM	pA_2_ (CL_95%_)
NPS	8.78 (8.22‐9.34)	1.00	
NPS(2‐20)	7.22 (6.62‐7.82)	1.14 ± 0.05	
NPS(3‐20)	crc incomplete		
NPS(1‐10)	6.55 (6.36‐6.74)	1.09 ± 0.14	
NPS(1‐6)	crc incomplete		
PWT1‐NPS	crc incomplete		
[Ala^7^]NPS	6.71 (6.38‐7.04)	1.03 ± 0.03	
[Bip^2^]NPS	7.04 (6.67‐7.41)	1.01 ± 0.04	
[Ala^3^]NPS	crc incomplete		
[D‐Ala^5^]NPS	7.23 (6.56‐7.90)	1.09 ± 0.04	
[Aib^5^]NPS	6.84 (6.48‐7.20)	0.99 ± 0.03	
[D‐Val^5^]NPS	Inactive		6.39 (6.01‐6.77)
[^*t*^Bu‐D‐Gly^5^]NPS	Inactive		6.48 (5.81‐7.15)
[D‐Pen‐S‐p(^*t*^BuBzl)^5^]NPS	Inactive		6.60 (5.96‐7.24)
SHA 68	Inactive		7.59 (7.05‐8.13)

The pharmacological parameters of all the NPSR ligands evaluated in the DMR assay performed in HEK293_mNPSR_ cells have been summarized in Table 2. In summary, NPS was the most potent NPSR agonist followed by NPS (2‐20), [Bip^2^]NPS, and [D‐Ala^5^]NPS which were approximately 30‐fold less potent, and NPS (1‐10), [Ala^7^]NPS, and [Aib^5^]NPS which were approximately 100‐fold less potent. The fragment NPS (1‐6) and the analogue [Ala^3^]NPS displayed a very low potency. NPS (3‐20) and PWT1‐NPS generated incomplete concentration‐response curves because of their lack of NPSR selectivity at high concentrations. SHA 68 was the most potent NPSR antagonist while peptide NPSR antagonists were approximately 10‐fold less potent.

## 4 DISCUSSION AND CONCLUSION

Preclinical studies suggest NPSR as a promising therapeutic target for the treatment of anxiety disorders, cognitive deficits, pain, and drug addiction.^5^ The identification of small molecule antagonists and, particularly, agonists selective for NPSR is now mandatory to proceed with clinical investigations of innovative drugs targeting this receptor. The establishment and optimization of reliable in vitro assays to investigate NPSR pharmacology will likely speed up the drug discovery process toward the identification of such molecules. As reported for several GPCRs, the intracellular signaling cascade that follows NPSR activation is complex and involves different parallel pathways such as Gq, Gs, and extracellular signal‐regulated kinases (ERKs)^1^, ^3^, ^34^, ^35^). Additionally, the complexity of NPSR signaling has been recently underlined by the identification of NPSR biased agonists, such as NPS (1‐10)^34^ and SFKN‐NH_2,_ 36 that preferentially signal through IP_3_‐DAG‐Ca^2+^ second messengers. This class of NPSR ligands may be useful as innovative anxiolytics.^36^, ^37^ Despite this NPSR signaling complexity, structure‐activity relationship studies and drug discovery programs targeting NPSR have been conducted so far only with classical single end‐point assays (for reviews see 4 and 5). This approach may be reductionist and incomplete. Thus, in the present study, the NPS signal nature and pharmacological profile of a large panel of NPSR ligands have been investigated at the recombinant murine NPSR using the DMR assay. This label‐free approach offers the opportunity to comprehensively evaluate, in a noninvasive manner, the action of molecules after receptor binding, thus providing a global view on receptor‐dependent cellular perturbations.^6^, ^7^

Neuropeptide S stimulated DMR responses in HEK293_mNPSR_ cells, but not in HEK293 cells, thus demonstrating that this signal is caused exclusively by the interaction of NPS with the NPSR receptor. The NPS‐evoked DMR signal was largely abolished by blocking the Gq pathway with FR900359, thus suggesting that the NPS DMR response in HEK293_mNPSR_ cells is predominantly Gq mediated. Of note, a recent study reported FR900359 as a potent inhibitor of the Gβγ‐mediated calcium influx,^38^ thus the presence of a Gβγ‐dependent component of the NPS signal cannot be excluded from our data. However, a small but significant Gq independent DMR signal was measured under these experimental conditions. This is different from the calcium mobilization assay where, as expected, FR900359 completely abolished all NPS effects. Thus, while the calcium mobilization assay only measures the Gq (and eventually Gβγ)‐dependent, ‐ component of NPSR signaling, the DMR assay is able to record a more comprehensive view cellular activity post NPSR activation. In the present study, we were not able to identify the mechanism underlying the FR900359‐resistant component of the NPS DMR signal. The experiments performed with PTX and rolipram excluded the involvement of Gi‐ and Gs‐related pathways. This was largely expected for PTX, since no data in the literature reported NPSR coupling to Gi, but not for rolipram since several papers showed an increase of cAMP levels after the activation of NPSR, thus suggesting that NPSR can couple to Gs proteins. Of note, the majority of these studies have been performed with cells expressing the human isoforms of the receptor.^3^, ^34^, ^35^ Possible differences in the coupling between the human and the murine NPSR may explain the discrepancy between our data that excludes a Gs dependent component of the NPSergic DMR response and those from literature reporting increase of cAMP levels in response to human NPSR activation. It is possible that other signaling pathways activated by NPSR, for example, the ERK pathway,^1^, ^34^ might be responsible for the Gq independent component of the DMR signal elicited by NPS in HEK293_mNPSR_ cells.

The pharmacological profile of NPSR in the DMR assay has been investigated using several NPSR ligands acting as full and partial agonists and pure antagonists in the calcium mobilization assay. The DMR effects of these compounds solely derive from their capability to activate NPSR since we selected their concentration range based on lack of DMR signal in wild‐type cells. Overall, the DMR pharmacological profile of NPSR looks similar but not identical to that reported in literature using the calcium mobilization assay. The first aspect that deserves attention is related to the efficacy of NPSR partial agonists. In fact, in calcium mobilization experiments, [Bip^2^]NPS, [D‐Ala^5^]NPS, [Aib^5^]NPS, and [Ala^3^]NPS behaved as partial agonists with α‐values in the range 0.4‐0.6.^23^, ^24^, ^25^ In contrast, in the present DMR experiments, all these NPS derivatives displayed maximal effects similar to that of NPS, thus behaving as full NPSR agonists. This difference can be explained considering that ligand efficacy is a strongly system‐dependent pharmacological parameter, that increases proportionally with increasing efficiency of the stimulus/response coupling.^39^ Most probably in the DMR assay, signal amplification phenomena due to integration of all cellular events that follow receptor activation, make the stimulus/response coupling particularly efficient therefore leading to a relative overestimation of ligand efficacy. Of note, similar considerations can be done for the compound [D‐Cys(^*t*^Bu)^5^]NPS, that behaves as a pure NPSR antagonist in the calcium mobilization assay,^26^ while in this study displayed robust residual agonist activity behaving as NPSR partial agonist.

As far as agonist potency is concerned, the rank order obtained in the present study was similar, although not identical, to that previously recorded in calcium mobilization studies. In particular, a good match was obtained with the following NPS ligands: NPS(2‐20), NPS(3‐20) and NPS(1‐6),^23^ [Bip^2^]NPS,^24^ [D‐Ala^5^]NPS, and [Aib^5^]NPS.^25^ Different potencies in the DMR and calcium assay were obtained with [Ala^3^]NPS, [Ala^7^]NPS and NPS(1‐10). In particular, NPS(1‐10) displayed similar potency in calcium studies as NPS and was defined as the minimum NPS fragment able to maintain the same in vitro pharmacological activity as the full‐length sequence.^23^ In the present study, NPS(1‐10) appeared 100‐fold less potent than NPS. Of note, NPS(1‐10) was recently defined by Liao et al as a calcium‐biased NPSR agonist.^34^ At present, we are unable to explain these differences in ligand potency between calcium mobilization and DMR assays. However, it should be underlined that these peptides were tested by Roth et al^23^ on the human NPSR, thus species‐specific receptor isoforms, rather than assay‐related differences, may eventually explain these discrepancies.

Regarding NPSR agonists, another point that must be addressed is the pharmacological behavior of PWT1‐NPS. This is a tetrabranched derivative of NPS that behaved in calcium experiments as an NPSR full agonist 3‐fold more potent than the natural peptide. The high in vitro potency of PWT1‐NPS was associated with a high in vivo potency and long‐lasting duration of action.^31^ These features seem to be common to PWT derivatives of other neuropeptides including nociceptin/orphanin FQ, opioids and tachykinins (reviewed in 40). In DMR experiments, we were not able to fully investigate the pharmacological profile of PWT1‐NPS, since at concentrations higher than 10 nmol L^−1^, the peptide produced off target effects, that is, eliciting DMR effects in wild‐type cells. Of note, the reduced selectivity of PWT1‐NPS was not detected in the calcium mobilization assay where selective NPSR antagonists displayed similar values of potency when challenged against NPS and PWT1‐NPS.^31^ These findings suggest that the DMR test can provide more exhaustive and complete information regarding ligand selectivity compared to classical assays. It is worth mentioning that Rizzi et al reported a reduced selectivity of PWT derivatives of nociceptin/orphanin FQ compared to the natural peptide in bioassay studies performed with tissues from receptor knockout mice,^41^ moreover PWT derivatives of nociceptin/orphanin FQ produced off‐target effects in DMR studies^20^; thus a certain loss of selectivity may be a common feature of tetrabranched peptide derivatives synthesized using the PWT technology and DMR can be useful approach to reveal this aspect.

Finally, the selective NPSR antagonists [D‐Val^5^]NPS, [^*t*^Bu‐D‐Gly^5^]NPS, [D‐Pen‐S‐p(^*t*^BuBzl)^5^]NPS, and SHA 68 were tested for their ability to counteract NPS‐evoked DMR signal. All compounds produced a dextral displacement of the concentration‐response curve to NPS with no modification of the agonist maximal effect, thus confirming the competitive type of interaction reported in previous studies.^27^, ^28^, ^29^, ^30^, ^32^, ^33^ The following rank order of antagonist potency has been obtained: SHA 68 > [D‐Pen‐S‐p(^*t*^BuBzl)^5^]NPS > [^*t*^Bu‐D‐Gly^5^]NPS > [D‐Val^5^]NPS, that perfectly matches previously described results in literature based on calcium mobilization studies (see references above). It should, however, be mentioned that in absolute terms the potency of NPSR antagonists estimated in the DMR assay was on average 3‐fold lower than that estimated in calcium mobilization studies. A similar trend has been obtained in our laboratory with a series of antagonists selective for the nociceptin/orphanin receptor and with naloxone on classical opioid receptors evaluated in the DMR^20^ and in calcium mobilization studies performed in cells expressing chimeric G proteins.^42^, ^43^, ^44^ Further studies are clearly needed to corroborate these initial findings and eventually to investigate the reasons underlying this tendency of the DMR assay to underestimate antagonist potency.

In conclusion, the results obtained in this study further corroborate the usefulness of the DMR assay for the investigation of the pharmacological profile of GPCRs, as well as their signaling properties. In particular, information from the present DMR studies complements data from previous calcium mobilization studies regarding pharmacological features that is, efficacy, potency, and selectivity of action of a large panel of NPSR ligands. The label‐free nature of the DMR assay associated with its high sensitivity will likely allow in the near future to perform studies in cell lines and eventually in primary culture cells expressing the native NPSR, thus substantially increasing the translational value of in vitro pharmacological studies.

## AUTHOR CONTRIBUTIONS

Participated in research design: CR and GC.

Conducted experiments: CR and FF.

Contributed new reagents or analytic tools: RG, EM, RKR, and DP.

Performed data analysis: CR and FF.

Wrote or contributed to the writing of the manuscript: CR, GC, RG, and RKR. The author thank Mark Bird (University of Leicester) for proofreading the article.

## CONFLICT OF INTEREST

All the authors declare no conflicts of interest.
